# Primary Immunodeficiency-Type Ataxia-Telangiectasia Revealed by Splenic Abscesses

**DOI:** 10.7759/cureus.64595

**Published:** 2024-07-15

**Authors:** Addou Bebana, Ayad Ghanam, Hassnae Tkak, Aziza Elouali, Abdeladim Babakhouya, Maria Rkain

**Affiliations:** 1 Department of Pediatrics, University Hospital Mohammed VI, Faculty of Medicine and Pharmacy, Mohammed First University, Oujda, MAR

**Keywords:** splenic abscesses, neoplasia, telangiectasia, ataxia, immune deficiency

## Abstract

Ataxia-telangiectasia (A-T) is a rare inherited autosomal recessive disease. It is associated with an alteration in the *ATM* gene, located on chromosome 11q22-23, which codes for a protein involved in a complex way in cell cycle regulation and cell protection. It is characterized by cerebellar ataxia, cutaneous and ocular telangiectasia, and an immune deficiency responsible for recurrent infections. Diagnosis is generally delayed due to the late onset of neurological symptoms and telangiectasia. People suffering from this condition are particularly sensitive to ionizing radiation, which considerably increases their risk of developing neoplasia. We report an observation of a primary immunodeficiency-type A-T revealed by recurrent fever and multiple splenic abscesses.

## Introduction

Ataxia-telangiectasia (A-T) is an autosomal recessive disorder. It is characterized by cerebellar ataxia, cutaneous and ocular telangiectasia, and severe immune deficiency. Patients with this condition are hypersensitive to ionizing radiation and have a high predisposition to cancer [1]. Despite its higher prevalence in children of European origin, A-T shows an increased incidence of neoplasia in black compared to white individuals [2]. Like any immune deficiency, A-T is known to be a breeding ground for infections. The particularity of our observation is that the diagnosis of A-T was revealed by splenic abscesses, which remain absent in our context.

A-T is a rare pathology in children, with an average age of discovery of three years. The prognosis of this disease is grave, largely due to the increased risk of infections and malignant complications. In the natural progression of the disease, life expectancy rarely exceeds 20 years [3].

## Case presentation

**Figure 1:**
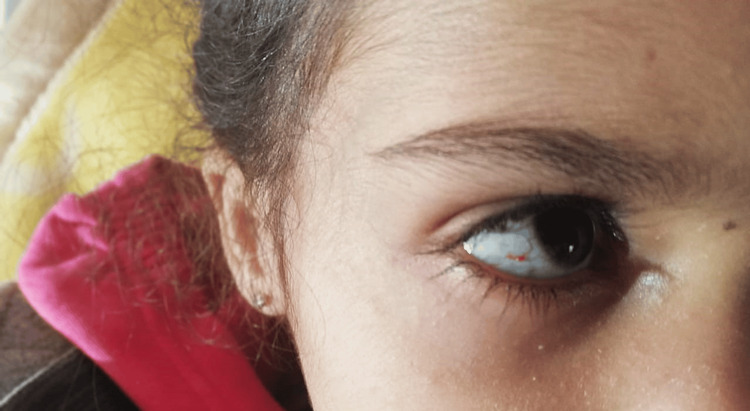
Ocular telangiectasia.

A five-year-old female child had previously presented with repeated episodes of pneumonia. She was admitted to the pediatric ward with a recurrent fever that had been evolving for two months, associated with a chronic cough. Clinical examination on admission revealed a fever, ataxic gait, and staturo-ponderal retardation at minus three standard deviations. Pulmonary auscultation revealed crackling rales. Abdominal examination revealed splenomegaly and a liver that was palpable 2 cm below the costal margin. Mucocutaneous examination revealed telangiectasia of the sclera (Figure 1).

**Figure 2:**
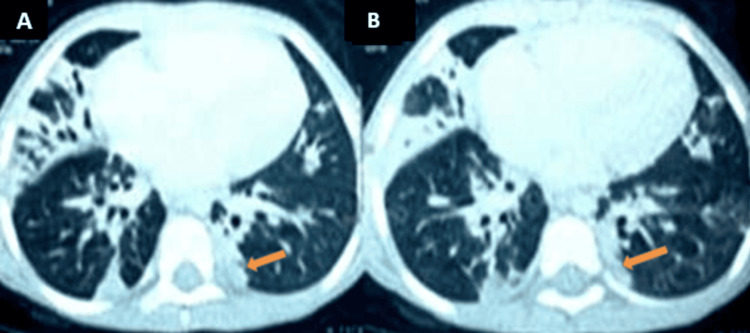
Thoracic CT scan (A and B) showing bilateral foci of alveolar condensation containing predominantly basal aerial bronchograms.

Initial workup showed inflammatory anemia with lymphopenia controlled on two blood counts of 590/mm^3^ and 960/mm^3^. Inflammatory syndrome was noted with a C-reactive protein of 58 mg/L, SV 110 mm. Blood culture was negative, diagnostic tests for tuberculosis (tuberculin intradermal reaction and BK test in gastric tubing fluid) and viral serologies (human immunodeficiency virus, hepatitis B virus, hepatitis C virus, cytomegalovirus) were negative, and bone marrow examination was normal. On radiological investigation, the chest CT showed bilateral alveolar condensation foci with air bronchograms predominantly at the basal level (Figure 2).

**Figure 3:**
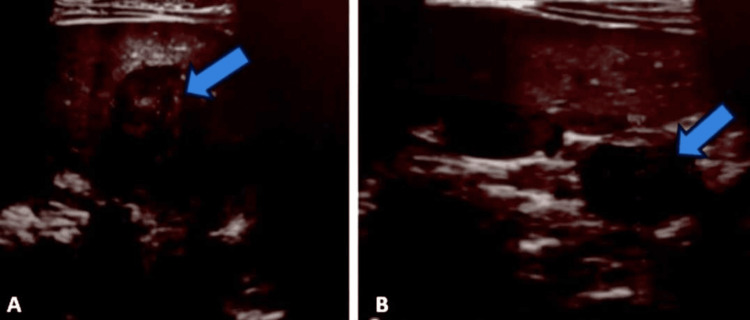
Abdominal ultrasound (A and B) showing splenomegaly with several well-limited, hypoechoic, heterogeneous, rounded formations containing isoechoic areas of variable size, with the largest measuring 32 × 24 mm.

**Figure 4:**
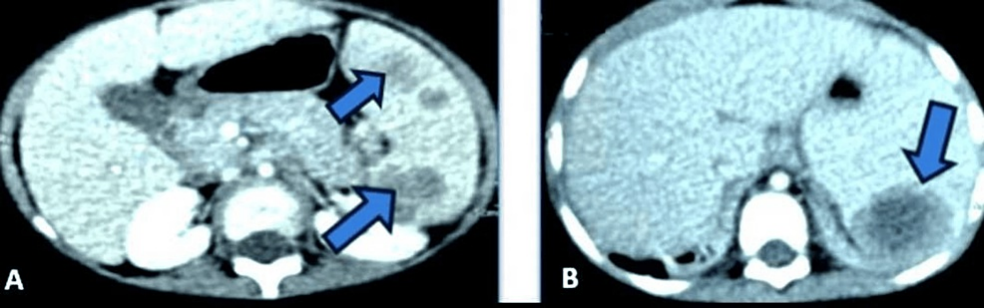
Thoraco-abdominal CT scan (A and B) showing the liver increased in size, with regular contours and homogeneous density, an enlarged spleen, heterogeneous, multiple lesion sites, well-limited, heterogeneous hypodense, necrotic, and heterogeneously enhancing after the injection of contrast medium.

Abdominal ultrasonography showed splenomegaly with several rounded, well-limited hypoechoic, heterogeneous formations containing hyperechoic and isoechoic areas of variable size, the largest measuring 32 × 24 mm, indicating multiple splenic abscesses, and homogeneous hepatomegaly (Figure 3). An abdominal CT scan revealed an enlarged liver with regular contours and homogeneous density, and an enlarged spleen with multiple, well-limited, hypodense, heterogeneous, necrotic lesions that enhanced heterogeneously after injection of contrast medium (PDC) in favor of multiple splenic abscesses (Figure 4).

Immunological tests showed IgG <3.2 g/L (normal = 5.5-10.2 g/L), IgA <0.25 g/L (normal = 0.46-1.5 g/L), IgM <0.42 g/L (normal = 0.54-1.53 g/L), and IgE <25 IU/mL (normal). The alpha-fetoprotein level was elevated to 138 ng/mL (normal = 0-7 ng/mL). Genetic testing was not performed. Based on these clinical, biological, radiological, and immunological findings, the diagnosis of A-T immune deficiency type was confirmed. The child was treated with probabilistic antibiotic therapy and immunoglobulin infusions, with a good clinical and radiological outcome.

## Discussion

A-T is an autosomal recessive disorder first described by Luis-Barr in 1941, characterized mainly by cerebellar degeneration, telangiectasia, immunodeficiency, cancer predisposition, and radiation sensitivity. Often referred to as a genome instability or DNA damage response syndrome, A-T exhibits its clinical manifestations.The global prevalence of A-T is estimated at between 1 in 40,000 and 1 in 100,000 live births [4]. Age at diagnosis is associated with the occurrence of ocular telangiectasia [5].

A literature review helped us focus on the clinical manifestations of this disease and the properties of the ATM protein, taking into account advances in our understanding of its pathophysiology. The diagnosis of A-T is generally based on a clinical triad characterized by neurological symptoms, notably a progressive static cerebellar syndrome, oculocutaneous manifestations such as telangiectasias or café-au-lait spots, and an immune deficiency involving both humoral and cellular components, leading to recurrent respiratory and pulmonary tract infections, with increased serum levels of alpha-fetoprotein [6].

The clinical presentation of A-T varies from child to child, which can delay diagnosis until school age, when neurological symptoms such as gait disorders, as well as telangiectasia, become apparent or worsen. Different forms or clinical presentations of A-T have been described, namely, the “classic,” “early,” or “childhood” form, while more attenuated forms are described as “variant” or “atypical” of late-onset. These two clinical presentations are different, but a widely accepted understanding of A-T indicates that individuals with an atypical form typically experience milder symptoms and a later onset of the disease [4].

Cerebellar ataxia is usually the first sign of neurodegeneration, often identified by a delay in walking around the age of two years. Subsequently, a range of kinetic cerebellar symptoms such as involuntary movements, diadochokinetic, and hypotonia complete the clinical picture, leading to loss of walking ability and bed dependence in adolescence, with repercussions on growth and body weight [6].

Telangiectasias, recognized as the second sign that can generally be seen around the age of four to six years, can often be observed but are not always present. Initially localized only to the eyes, and located symmetrically on the bulbar conjunctivae, ocular telangiectasias are often a reason for ophthalmology consultation [7].

Later, cutaneous telangiectasias appear mainly around the ears. Other skin signs may also be present, such as cafe-au-lait macules, vitiligo, early skin atrophy in exposed areas, premature appearance of white hair, and warty lesions [1,8].

Immune deficiency in A-T is often mixed in nature, involving both cellular and humoral deficiencies, and tends to become progressively more severe [6,9]. Cellular deficiency involves CD4 and CD8 lymphocytes and results in a negative intradermal reaction to tuberculin and a negative mitogen lymphocyte proliferation test [10,11]. Humoral deficiency mainly affects IgA immunoglobulins and IgG subclasses [6]. The overall IgG level may be normal, but it is essential to systematically supplement this assay with that of the subclasses. Indeed, a compensatory increase in IgG subclasses may mask a severe deficiency in a specific subclass, or an IgG level not increased in the event of infection [12]. In addition, IgG2 or IgG4 deficiencies may not be detectable in the overall IgG level if they are not accompanied by IgG1 deficiency. Occasionally, an increase in IgM is observed [13].

Patients with A-T show increased sensitivity to ionizing radiation. The first children diagnosed with A-T and treated with radiotherapy experienced serious complications [10]. It has also been found that A-T patients tend to develop cancers, partly due to increased sensitivity to radiation, but mainly due to acquired chromosomal abnormalities [14].

The immune deficiency associated with A-T often leads to respiratory problems, which are frequently detected before the onset of neurological symptoms [15]. In studies, pulmonary disorders such as recurrent bronchial infections, localized or generalized infectious pneumopathies, pleuro-pneumonia, bronchiectasis, episodes of hemoptysis, and sometimes chronic interstitial syndrome are frequent. Nevertheless, bronchopulmonary superinfections remain the most frequent complication of A-T-associated lung disease. The most frequently identified germs are encapsulated bacteria such as *Haemophilus influenzae* or *Streptococcus pneumonia*, as well as *Pseudomonas aeruginosa* [13].

In our case, the patient presented with recurrent fever and chronic cough, and radiological examination revealed multiple splenic abscesses, a complication of A-T.

To prevent potential difficulties that can affect A-T patients, respiratory management is recommended, including regular functional monitoring and minimizing radiological examinations. Follow-up should be careful, particularly concerning the risk of tumor development, requiring regular clinical and ultrasound monitoring. In cases of humoral immune deficiency, replacement therapy with polyvalent immunoglobulins may be considered.

## Conclusions

A-T is a serious genetic disease with multi-systemic expression, whose prognosis depends mainly on pulmonary complications, increased risk of infection, and malignant complications. As no specific treatment is currently available, it is important to prevent infection by antibiotic prophylaxis and avoiding radiation exposure wherever possible.

Our case highlights a rare presentation of A-T, with the occurrence of multiple splenic abscesses, a complication scarcely documented in the medical literature. Although respiratory problems are common manifestations of this disease, it is crucial to remain alert to other atypical presentations, such as this one. This observation underlines the importance of a comprehensive clinical approach in the diagnosis and management of patients with A-T to detect early potentially serious but rare complications such as splenic abscesses.
